# Synthesis, crystal structure and photophysical properties of bis­[2,6-di­fluoro-3-(pyridin-2-yl)pyridine-κ*N*](tri­fluoro­methane­sulfonato-κ*O*)silver(I)

**DOI:** 10.1107/S2056989021011282

**Published:** 2021-11-04

**Authors:** Suk-Hee Moon, Sanghyun Paek, Youngjin Kang

**Affiliations:** a Department of Food and Nutrition, Kyungnam College of Information and Technology, Busan 47011, Republic of Korea; b Department of Chemistry & Energy Engineering, Sangmyung University, Seoul 03016, Republic of Korea; c Division of Science Education, Kangwon National University, Chuncheon 24341, Republic of Korea

**Keywords:** crystal structure, 2,3′-bi­pyridine, silver complex, luminescence, OLED

## Abstract

In the title compound, [Ag(CF_3_SO_3_)(C_10_H_6_F_2_N_2_)_2_], the Ag^I^ centre adopts a highly distorted trigonal–planar coordination environment resulting from its coordination by one O atom of the tri­fluoro­methane­sulfonate anion and the pyridine N atoms of two crystallographically independent 2′,6′-di­fluoro-2,3′-bi­pyridine ligands, which display very similar conformations to one another. Pairwise Ag⋯O–SO_2_CF_3_^−^ [Ag⋯O = 2.8314 (14) Å] inter­actions and inter­molecular C—H⋯O inter­actions between inversion-related units lead to the formation of an eight-membered cyclic dimer in which the silver atoms are separated by 6.2152 (3) Å. In the crystal, the dimers are linked through C—H⋯O hydrogen bonds, halogen⋯π and weak π–π stacking inter­actions, resulting in the formation of a three-dimensional supra­molecular network. The title compound exhibits a strong and broad emission band from 400 nm to 550 nm in solution and its photoluminescence quantum efficiency is estimated to be *ca* 0.2, indicating that the title compound could have applications as an emitting material in organic light-emitting diodes (OLEDs).

## Abstract 2

The Ag^I^ atom in the title compound, which exhibits strong blue emission, adopts a highly distorted trigonal–planar geometry coordinated by two pyridine N atoms of two crystallographically independent 2′,6′-di­fluoro-2,3′-bi­pyridine ligands and one O atom of the tri­fluoro­metane­sulfonate anion.

## Chemical context

Recently, great attention has been paid to 2,3′-bi­pyridine-based Ir^III^ and Pt^II^ complexes by many researchers because of their applicability to OLEDs and solid-state lighting (Kang *et al.*, 2021 ▸; Reddy & Bejoymohandas, 2016 ▸). In particular, 2′,6′-di­fluoro-2,3′-bi­pyridine complexes of iridium(III) are considered to be strong candidates as both blue triplet emitters in phospho­rescent organic light-emitting diodes (PHOLEDs) and single dopants in white organic light-emitting diodes (WOLEDs) (Zaen *et al.*, 2019 ▸; Kang *et al.*, 2020 ▸; Lee *et al.*, 2018 ▸). Despite these investigations, reports regarding the structures and photoluminescence properties of 2,3′-bi­pyridine-based group-11 metal complexes are scarce, and related research is limited (Li *et al.*, 2019 ▸). Among the group-11 elements, coordination polymers of Ag^I^ have been demonstrated to exhibit structural diversity as a result of the *d* ^10^ configuration of the metal ion (Lee *et al.*, 2020 ▸). Moreover, the various coordination environments around the Ag^I^ centre are generally constructed by the ligands, solvent mol­ecules, and counter-anions (Lee *et al.*, 2016 ▸). Until now, there has been no report with respect to an Ag^I^ complex bearing a 2′,6′-di­fluoro-2,3′-bi­pyridine ligand as compared to 2,2-bi­pyridine-based Ag^I^ complexes (Pal *et al.*, 2020 ▸). This fact prompted us to investigate the structures and luminescent properties of 2,3′-bi­pyridine-based Ag^I^ complexes: in this study, we report the preparation, structural characterization and luminescent properties of an Ag^I^ complex of 2′,6′-di­fluoro-2,3′-bi­pyridine. 
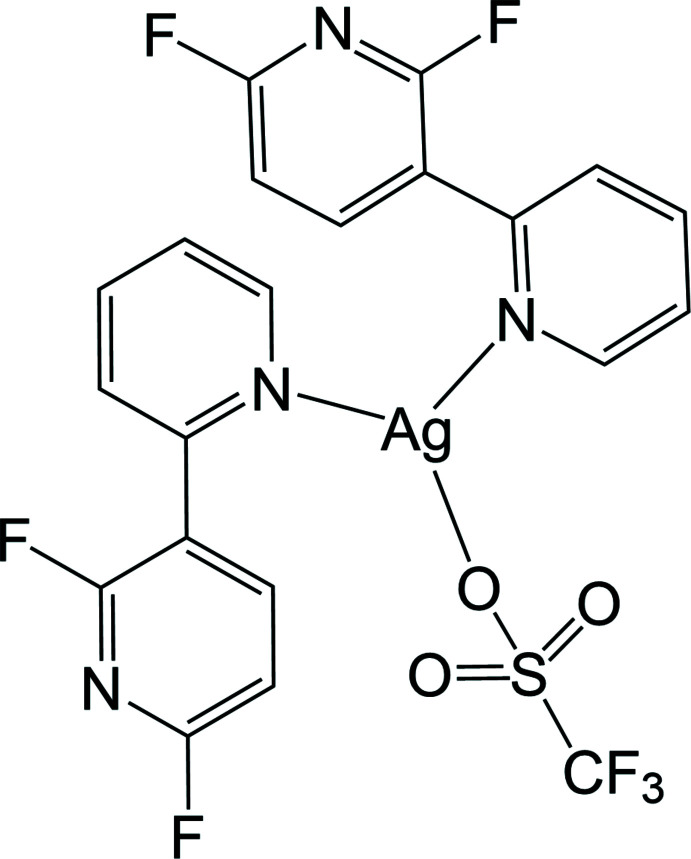


## Structural commentary

**Figure 1:**
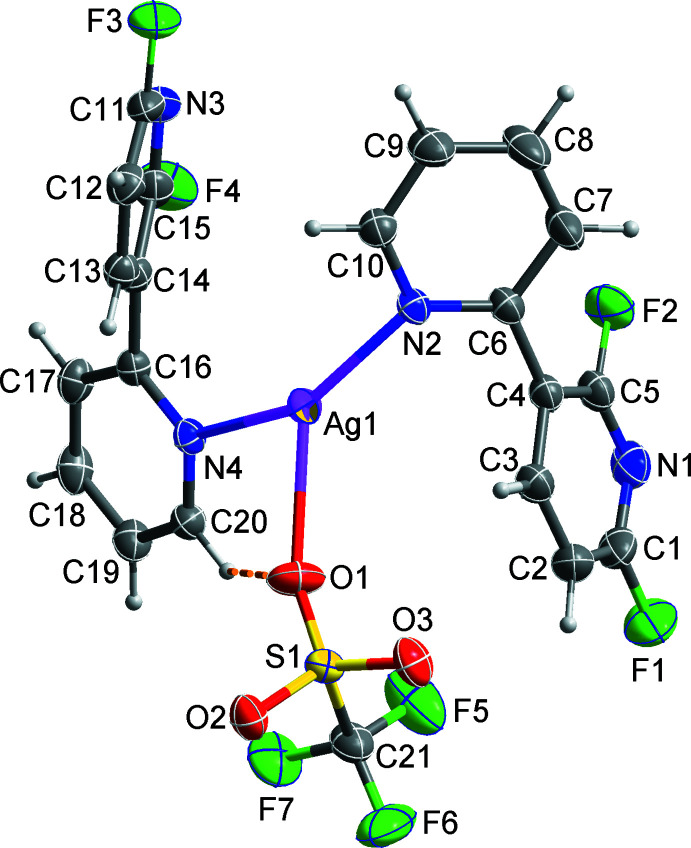
The mol­ecular structure of the title compound, showing displacement ellipsoids at the 50% probability level. The orange dashed line represents an intra­molecular C—H⋯O inter­action.

**Figure 2:**
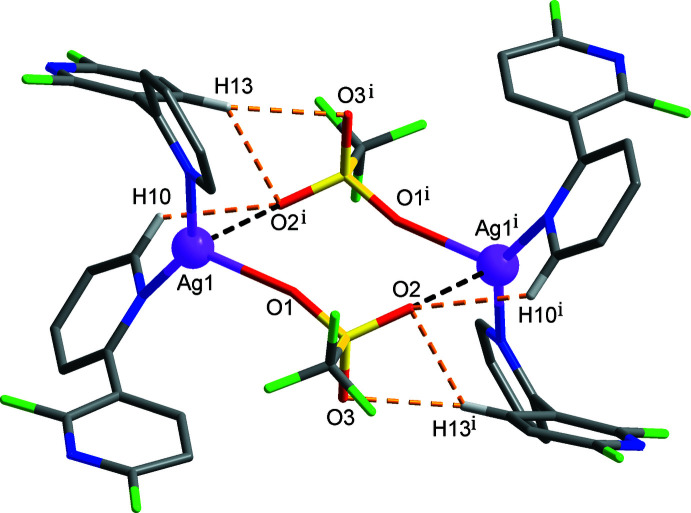
Dimeric structure formed by Ag⋯O (black dashed lines) and C—H⋯O (yellow dashed lines) inter­actions [symmetry code: (i) −*x* + 1, −*y*, −*z* + 1]. Atom colours: violet = silver, yellow = sulfur, green = fluorine, red = oxygen, blue = nitro­gen, grey = carbon and white = hydrogen.

The asymmetric unit in the title compound consists of an Ag^I^ cation, a CF_3_SO_3_^−^ tri­fluoro­methane­sulfonate anion and two crystallographically independent C_10_H_6_F_2_N_2_ 2′,6′-di­fluoro-2,3′-bi­pyridine ligands, which adopt very similar conformations, such that the dihedral angles between the pyridyl rings in the N1- and N3-containing mol­ecules are 53.11 (5) and 53.10 (7)°, respectively. As shown in Fig. 1 ▸, the Ag^I^ ion is coordinated by two pyridine N atoms (N2 and N4) from two 2′,6′-di­fluoro-2,3′-bi­pyridine ligands and one O atom from the tri­fluoro­methane­sulfonate anion, forming a highly distorted trigonal–planar geometry. Selected bond lengths and angles around the Ag1 atom are given in Table 1 ▸: the N—Ag—N and N—Ag—O angles fall in the range 86.55 (5)–148.65 (5)°, deviating significantly from an ideal trigonal–planar geometry. This may reflect the influence of an additional Ag⋯O–SO_2_CF_3_^−^ inter­action between the metal ion and an O atom of an adjacent tri­fluoro­methane­sulfonate anion [Ag1⋯O2^i^ = 2.8314 (14) Å; black dashed lines in Fig. 2 ▸; symmetry code: (i) −*x* + 1, −*y*, −*z* + 1]. The Ag^I^ atom is displaced out of the trigonal N2, N4, O1 coordination plane by 0.1057 (9) Å. The C6–C10/N2 and C16–C20/N4 pyridine rings coordinated to the Ag^I^ centre are tilted by 25.75 (10)° with respect to each other. The pairwise Ag⋯O links lead to the formation of an eight-membered [Ag—O—S—O—]_2_ cyclic dimer, in which the silver atoms are separated by 6.2152 (3) Å. The cyclic dimer is consolidated by C—H⋯O inter­actions (Table 2 ▸; yellow dashed lines in Fig. 2 ▸).

## Supra­molecular features

**Figure 3:**
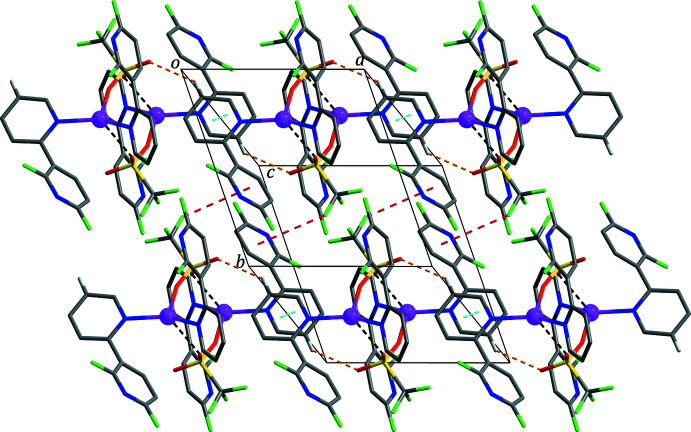
The two-dimensional supra­molecular network formed through C—H⋯O hydrogen bonds (yellow dashed lines), F⋯π (red dashed lines) and π–π stacking (sky-blue dashed lines) inter­actions. For clarity, H atoms not involved in the inter­molecular inter­actions have been omitted. Atom colours as in Fig. 2 ▸.

**Figure 4:**
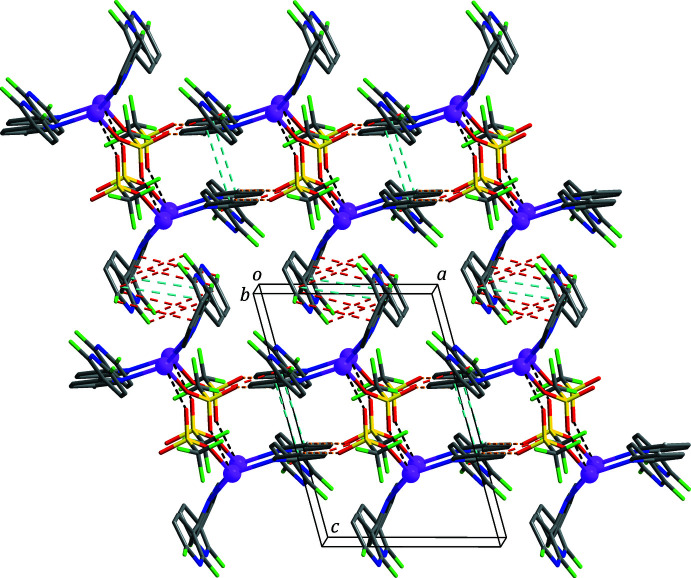
The three-dimensional supra­molecular network formed through F⋯π (red dashed lines) and π–π stacking (sky-blue dashed lines) inter­actions. For clarity, H atoms not involved in the inter­molecular inter­actions have been omitted. Atom colours as in Fig. 2 ▸.

In the extended structure, the dimers are linked through C19—H19⋯O3 hydrogen bonds (Table 2 ▸) and weak π–π stacking inter­actions [yellow and sky-blue dashed lines in Fig. 3 ▸, respectively; *Cg*4⋯*Cg*4^ii^ = 3.9737 (11) Å; *Cg*4 is the centroid of the C16–C20/N4 ring; symmetry code: (ii) −*x*, −*y*, −*z* + 1] between the pyridine rings, forming a chain structure propagating along the *a*-axis direction. Neighbouring chains are connected by halogen⋯π inter­actions [red dashed lines in Fig. 3 ▸; F6⋯*Cg*3^iii^ = 3.06 (2) Å; *Cg*3 is the centroid of the C11–C15/N3 ring; symmetry code: (iii) *x* + 1, *y* − 1, *z*], thereby generating a two-dimensional supra­molecular network lying parallel to the *ab* plane. Finally, these networks are stacked along the *c*-axis direction and connected by halogen⋯π and weak π–π stacking inter­actions [red and sky-blue dashed lines in Fig. 4 ▸, respectively; F1⋯*Cg*2^iv^ = 3.974 (2) Å; F2⋯*Cg*1^iv^ = 3.1424 (19) Å; *Cg*1⋯*Cg*1^iv^ = 4.2435 (13) Å; *Cg*1 and *Cg*2 are the centroids of the C1–C5/N1 and C6–C10/N2 rings, respectively; symmetry code: (iv) −*x* + 1, −*y*, −*z*], resulting in the formation of a three-dimensional supra­molecular network.

## Luminescent properties

**Figure 5:**
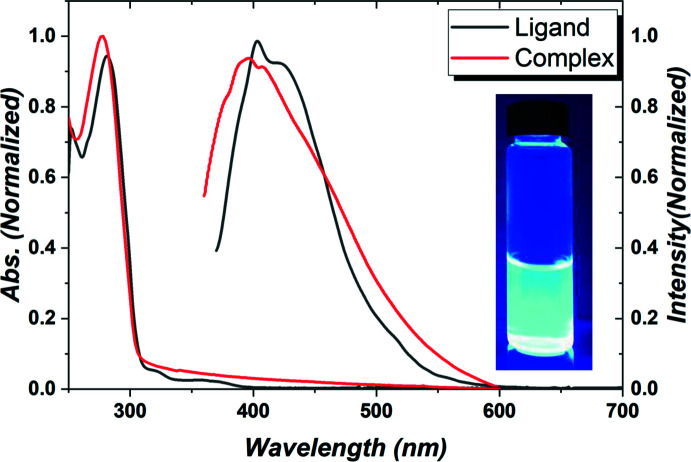
Absorption and emission spectra of the free ligand and the title compound in solution [concentrations = 1.0 × 10^−5^ *M*] at room temperature (inset: emission photo); ɛ ≃ 100,000 *M* ^−1^ cm^−1^.

In CH_2_Cl_2_ solution, the title compound exhibits a strong and broad emission band with *λ*_max_ = 400 nm, as shown in Fig. 5 ▸. This emission band may arise from π–π* transitions of the bi­pyridine ligand because the absorption of the title compound is very similar to that of the free ligand. Inter­estingly, upon the complexation of ligand with the Ag(CF_3_SO_3_) unit, significant blue-shifted emissions (> 50 nm) are observed as compared with bi­pyridine based Ir^III^ complexes (Lee *et al.*, 2009 ▸). Moreover, a broad emission from 400 nm to 500 nm in the title compound may be due to the predominantly fluorescent emission from the 2′,6′-di­fluoro-2,3′-bi­pyridine ligand because the emission maximum of the free ligand, *i.e*. phospho­rescent emission, occurs at approximately 450 nm (triplet energy, *T*_1_ = 2.82 eV). The observed emission of the title compound is therefore attributed to ligand-centered π–π* transitions with a minor contribution of an Ag-based metal-to-ligand charge-transfer transition. Similar dual-emission behaviour has been noted for some Ag^I^ complexes with 2-methyl­thio­thia­zole (Rogovoy *et al.*, 2019 ▸) and pyridyl­phosphine ligands (Baranov *et al.*, 2019 ▸). The emission intensity of the title compound was also higher than that of free ligand, as shown in Fig. 5 ▸. The photoluminescence quantum efficiency of the title compound was estimated to be *ca* 0.2 (Fig. 5 ▸, inset). Such an efficiency is large enough to potentially use the title compound as the emitting material in an organic light-emitting diode (OLED) application.

## Database survey

A survey of *SciFinder* (SciFinder, 2021 ▸) for transition-metal complexes bearing the 2′,6′-di­fluoro-2,3′-bi­pyridine moiety as a ligand gave 25 hits. They include reports about the crystal structures and photophysical properties of Ir^III^ and Pt^II^ complexes based on this ligand (HOVHAC, Lee *et al.*, 2009 ▸; OHUMUB01, Lee *et al.*, 2015 ▸; JUDZAL, Park *et al.*, 2015 ▸). The survey revealed no exact matches for the reported structure of the title complex. To the best of our knowledge, this is the first crystal structure reported for a silver complex with the title ligand.

## Synthesis and crystallization

All experiments were performed under a dry N_2_ atmosphere using standard Schlenk techniques. All solvents used in this study were freshly distilled over appropriate drying reagents prior to use. All starting materials were purchased commercially and used without further purification. The ^1^H NMR spectrum was recorded on a JEOL 400 MHz spectrometer. The ligand, 2′,6′-di­fluoro-2,3′-bi­pyridine (Park *et al.*, 2015 ▸) was synthesized according to the previous report. The title compound was synthesized as follows: the ligand (0.075 g, 0.39 mmol) in THF (2 ml) was added to Ag(CF_3_SO_3_) (0.47 g, 1.0 mmol) in MeOH (2 ml) in the dark at room temperature and the mixture was stirred for 10 min. After that, the mixture was slowly evaporated in the air and a dark environment to obtain crystals suitable for X-ray crystallographic analysis. ^1^H NMR (400 MHz, CD_3_CN) *δ* 8.67 (*d*, *J* = 4.4 Hz, 1H), 8.62 (*td*, *J* = 8.8, 1.2 Hz, 1H), 7.88–7.80 (*m*, 2H), 7.37–7.34 (*m*, 1H), 7.0.8 (*dd*, *J* = 9.2, 3.6 Hz, 1H). ^19^F NMR (376 MHz, CD_3_CN) *δ* −69.7, −71.8, 79.1. Analysis calculated for C_21_H_12_F_7_N_4_O_3_SAg: C 39.33; H 1.89; N 8.74%; found: C 39.44, H 1.86, N 8.70%.

## Refinement

Crystal data, data collection and structure refinement details are summarized in Table 3 ▸. All H atoms were positioned geometrically and refined using a riding model: C—H = 0.95 Å with *U*_iso_(H) = 1.2*U*_eq_(C).

## Supplementary Material

Crystal structure: contains datablock(s) I, New_Global_Publ_Block. DOI: 10.1107/S2056989021011282/hb7990sup1.cif

Structure factors: contains datablock(s) I. DOI: 10.1107/S2056989021011282/hb7990Isup2.hkl

CCDC reference: 2118061

Additional supporting information:  crystallographic information; 3D view; checkCIF report

## Figures and Tables

**Table 1:** Selected geometric parameters (Å, °)

Ag1—N2	2.2305 (14)	Ag1—O1	2.4879 (13)
Ag1—N4	2.2496 (14)		
			
N2—Ag1—N4	148.65 (5)	N4—Ag1—O1	86.55 (5)
N2—Ag1—O1	124.02 (5)		

**Table 2:** Hydrogen-bond geometry (Å, °)

*D*—H⋯*A*	*D*—H	H⋯*A*	*D*⋯*A*	*D*—H⋯*A*
C10—H10⋯O2^i^	0.95	2.60	3.239 (2)	125
C13—H13⋯O2^i^	0.95	2.60	3.173 (2)	119
C13—H13⋯O3^i^	0.95	2.50	3.400 (2)	159
C19—H19⋯O3^ii^	0.95	2.53	3.294 (2)	137
C20—H20⋯O1	0.95	2.55	3.197 (2)	126

Symmetry codes: (i) -x+1, -y, -z+1; (ii) x-1, y, z.

**Table 3:** Experimental details

Crystal data	
Chemical formula	[Ag(CF_3_O_3_S)(C_10_H_6_F_2_N_2_)_2_]
*M*_r_	641.28
Crystal system, space group	Triclinic, *P*\overline{1}
Temperature (K)	193
*a*, *b*, *c* (Å)	9.0627 (2), 10.9637 (3), 12.5727 (3)
α, β, γ (°)	82.4508 (11), 73.7215 (11), 71.5490 (11)
*V* (Å^3^)	1136.26 (5)
*Z*	2
Radiation type	Mo *K*α
μ (mm^−1^)	1.07
Crystal size (mm)	0.38 × 0.33 × 0.32

Data collection	
Diffractometer	Bruker APEXII CCD
Absorption correction	Multi-scan (*SADABS*; Bruker, 2014 ▸)
*T*_min_, *T*_max_	0.610, 0.746
No. of measured, independent and observed [*I* > 2σ(*I*)] reflections	19973, 5623, 5262
*R*_int_	0.029
(sin θ/λ)_max_ (Å^−1^)	0.668

Refinement	
*R*[*F* ^2^ > 2σ(*F* ^2^)], *wR*(*F* ^2^), *S*	0.025, 0.067, 1.12
No. of reflections	5623
No. of parameters	334
H-atom treatment	H-atom parameters constrained
Δρ_max_, Δρ_min_ (e Å^−3^)	0.33, −0.76

Computer programs: *APEX2* and *SAINT* (Bruker, 2014 ▸), *SHELXS97* and *SHELXTL* (Sheldrick, 2008 ▸), *SHELXL2014/7* (Sheldrick, 2015 ▸), *DIAMOND* (Brandenburg, 2010 ▸) and *publCIF* (Westrip, 2010 ▸).

## supplementary crystallographic information

### Crystal data

**Table:**

[Ag(CF_3_O_3_S)(C_10_H_6_F_2_N_2_)_2_]	*Z* = 2
*M**_r_* = 641.28	*F*(000) = 632
Triclinic, *P*1	*D*_x_ = 1.874 Mg m^−^^3^
*a* = 9.0627 (2) Å	Mo *K*α radiation, λ = 0.71073 Å
*b* = 10.9637 (3) Å	Cell parameters from 9858 reflections
*c* = 12.5727 (3) Å	θ = 2.5–28.3°
α = 82.4508 (11)°	µ = 1.07 mm^−^^1^
β = 73.7215 (11)°	*T* = 193 K
γ = 71.5490 (11)°	Block, colourless
*V* = 1136.26 (5) Å^3^	0.38 × 0.33 × 0.32 mm

### Data collection

**Table:**

Bruker APEXII CCD diffractometer	5262 reflections with *I* > 2σ(*I*)
φ and ω scans	*R*_int_ = 0.029
Absorption correction: multi-scan (SADABS; Bruker, 2014)	θ_max_ = 28.3°, θ_min_ = 2.5°
*T*_min_ = 0.610, *T*_max_ = 0.746	*h* = −12→11
19973 measured reflections	*k* = −14→14
5623 independent reflections	*l* = −16→14

### Refinement

**Table:**

Refinement on *F*^2^	Primary atom site location: structure-invariant direct methods
Least-squares matrix: full	Hydrogen site location: inferred from neighbouring sites
*R*[*F*^2^ > 2σ(*F*^2^)] = 0.025	H-atom parameters constrained
*wR*(*F*^2^) = 0.067	*w* = 1/[σ^2^(*F*_o_^2^) + (0.0356*P*)^2^ + 0.2813*P*] where *P* = (*F*_o_^2^ + 2*F*_c_^2^)/3
*S* = 1.12	(Δ/σ)_max_ = 0.001
5623 reflections	Δρ_max_ = 0.33 e Å^−^^3^
334 parameters	Δρ_min_ = −0.76 e Å^−^^3^
0 restraints	

### Special details

**Table:**

Geometry. All esds (except the esd in the dihedral angle between two l.s. planes) are estimated using the full covariance matrix. The cell esds are taken into account individually in the estimation of esds in distances, angles and torsion angles; correlations between esds in cell parameters are only used when they are defined by crystal symmetry. An approximate (isotropic) treatment of cell esds is used for estimating esds involving l.s. planes.

### Fractional atomic coordinates and isotropic or equivalent isotropic displacement parameters (Å^2^)

**Table:**

	*x*	*y*	*z*	*U*_iso_*/*U*_eq_	
Ag1	0.39687 (2)	0.11579 (2)	0.28356 (2)	0.02967 (5)	
S1	0.58088 (5)	−0.20353 (4)	0.43046 (4)	0.02529 (9)	
O1	0.47418 (19)	−0.09076 (14)	0.39174 (14)	0.0444 (4)	
O2	0.54144 (16)	−0.22732 (14)	0.54886 (11)	0.0356 (3)	
O3	0.74849 (17)	−0.22309 (15)	0.37857 (12)	0.0400 (3)	
C21	0.5358 (3)	−0.3333 (2)	0.38115 (18)	0.0392 (4)	
F5	0.5511 (2)	−0.31983 (18)	0.27233 (12)	0.0745 (5)	
F6	0.6317 (2)	−0.44672 (13)	0.40282 (16)	0.0701 (5)	
F7	0.38549 (17)	−0.33477 (14)	0.42807 (13)	0.0562 (4)	
F1	0.7648 (2)	−0.33112 (13)	−0.01970 (14)	0.0698 (5)	
F2	0.63399 (17)	0.08741 (13)	−0.11751 (10)	0.0460 (3)	
N1	0.7033 (2)	−0.12143 (18)	−0.06736 (14)	0.0414 (4)	
N2	0.57260 (17)	0.21084 (14)	0.17341 (12)	0.0249 (3)	
C1	0.7501 (3)	−0.21237 (19)	0.00565 (19)	0.0430 (5)	
C2	0.7813 (3)	−0.1947 (2)	0.10241 (18)	0.0414 (5)	
H2	0.8198	−0.2652	0.1500	0.050*	
C3	0.7538 (2)	−0.06916 (18)	0.12668 (16)	0.0334 (4)	
H3	0.7695	−0.0515	0.1940	0.040*	
C4	0.7031 (2)	0.03212 (17)	0.05317 (14)	0.0277 (3)	
C5	0.6836 (2)	−0.00397 (19)	−0.04231 (15)	0.0324 (4)	
C6	0.6718 (2)	0.16833 (17)	0.07537 (14)	0.0274 (3)	
C7	0.7452 (3)	0.2481 (2)	−0.00157 (18)	0.0443 (5)	
H7	0.8157	0.2158	−0.0700	0.053*	
C8	0.7154 (3)	0.3738 (2)	0.0216 (2)	0.0518 (6)	
H8	0.7646	0.4294	−0.0304	0.062*	
C9	0.6128 (3)	0.4179 (2)	0.12161 (18)	0.0408 (5)	
H9	0.5885	0.5049	0.1393	0.049*	
C10	0.5462 (2)	0.33348 (18)	0.19542 (16)	0.0313 (4)	
H10	0.4783	0.3635	0.2652	0.038*	
F3	0.13496 (19)	0.67539 (12)	0.27171 (13)	0.0583 (4)	
F4	−0.07604 (18)	0.40180 (14)	0.17706 (12)	0.0545 (4)	
N3	0.0298 (2)	0.53662 (16)	0.22662 (15)	0.0401 (4)	
N4	0.14448 (16)	0.10066 (14)	0.33810 (12)	0.0244 (3)	
C11	0.1051 (2)	0.56069 (17)	0.29307 (18)	0.0381 (4)	
C12	0.1546 (2)	0.48149 (18)	0.37758 (17)	0.0348 (4)	
H12	0.2070	0.5058	0.4234	0.042*	
C13	0.1239 (2)	0.36367 (17)	0.39263 (15)	0.0280 (3)	
H13	0.1566	0.3044	0.4499	0.034*	
C14	0.04554 (19)	0.33106 (16)	0.32476 (14)	0.0259 (3)	
C15	0.0017 (2)	0.42467 (19)	0.24485 (16)	0.0337 (4)	
C16	0.0162 (2)	0.20399 (16)	0.33730 (14)	0.0252 (3)	
C17	−0.1363 (2)	0.19243 (19)	0.35044 (16)	0.0327 (4)	
H17	−0.2251	0.2670	0.3494	0.039*	
C18	−0.1577 (2)	0.0716 (2)	0.36507 (17)	0.0356 (4)	
H18	−0.2613	0.0619	0.3747	0.043*	
C19	−0.0260 (2)	−0.03498 (18)	0.36549 (16)	0.0333 (4)	
H19	−0.0369	−0.1194	0.3747	0.040*	
C20	0.1222 (2)	−0.01603 (17)	0.35223 (15)	0.0289 (3)	
H20	0.2124	−0.0894	0.3532	0.035*	

### Atomic displacement parameters (Å^2^)

**Table:**

	*U*^11^	*U*^22^	*U*^33^	*U*^12^	*U*^13^	*U*^23^
Ag1	0.02256 (8)	0.02807 (8)	0.03687 (9)	−0.01100 (5)	−0.00184 (5)	0.00012 (5)
S1	0.0262 (2)	0.02496 (19)	0.0277 (2)	−0.00994 (16)	−0.01063 (16)	0.00272 (15)
O1	0.0493 (9)	0.0297 (7)	0.0602 (10)	−0.0111 (6)	−0.0309 (8)	0.0128 (7)
O2	0.0324 (7)	0.0467 (8)	0.0274 (7)	−0.0118 (6)	−0.0074 (5)	−0.0002 (6)
O3	0.0313 (7)	0.0560 (9)	0.0356 (7)	−0.0211 (6)	−0.0045 (6)	−0.0003 (6)
C21	0.0471 (11)	0.0352 (10)	0.0392 (11)	−0.0220 (9)	−0.0053 (9)	−0.0027 (8)
F5	0.1206 (15)	0.0919 (12)	0.0389 (8)	−0.0719 (12)	−0.0136 (9)	−0.0112 (8)
F6	0.0722 (10)	0.0270 (6)	0.1019 (14)	−0.0094 (7)	−0.0113 (9)	−0.0083 (7)
F7	0.0522 (8)	0.0609 (9)	0.0689 (10)	−0.0393 (7)	−0.0093 (7)	−0.0056 (7)
F1	0.0974 (13)	0.0359 (7)	0.0692 (10)	−0.0239 (8)	0.0022 (9)	−0.0199 (7)
F2	0.0640 (8)	0.0479 (7)	0.0289 (6)	−0.0161 (6)	−0.0189 (6)	0.0037 (5)
N1	0.0518 (11)	0.0434 (10)	0.0307 (9)	−0.0190 (8)	−0.0027 (7)	−0.0116 (7)
N2	0.0255 (7)	0.0279 (7)	0.0233 (7)	−0.0113 (6)	−0.0053 (5)	−0.0013 (5)
C1	0.0499 (12)	0.0298 (10)	0.0431 (12)	−0.0127 (9)	0.0041 (9)	−0.0126 (8)
C2	0.0435 (11)	0.0324 (10)	0.0394 (11)	−0.0061 (8)	−0.0046 (9)	0.0026 (8)
C3	0.0355 (10)	0.0351 (9)	0.0281 (9)	−0.0097 (8)	−0.0075 (7)	0.0003 (7)
C4	0.0286 (8)	0.0309 (8)	0.0229 (8)	−0.0108 (7)	−0.0028 (6)	−0.0022 (7)
C5	0.0373 (10)	0.0373 (10)	0.0228 (8)	−0.0135 (8)	−0.0047 (7)	−0.0017 (7)
C6	0.0299 (8)	0.0311 (8)	0.0232 (8)	−0.0128 (7)	−0.0061 (7)	−0.0003 (6)
C7	0.0578 (13)	0.0455 (11)	0.0290 (10)	−0.0285 (10)	0.0060 (9)	−0.0039 (8)
C8	0.0740 (16)	0.0464 (12)	0.0401 (12)	−0.0392 (12)	0.0005 (11)	0.0017 (10)
C9	0.0546 (13)	0.0319 (9)	0.0409 (11)	−0.0217 (9)	−0.0095 (9)	−0.0024 (8)
C10	0.0337 (9)	0.0309 (9)	0.0317 (9)	−0.0126 (7)	−0.0072 (7)	−0.0046 (7)
F3	0.0732 (10)	0.0287 (6)	0.0657 (9)	−0.0207 (6)	−0.0007 (7)	0.0006 (6)
F4	0.0618 (9)	0.0655 (9)	0.0485 (8)	−0.0220 (7)	−0.0370 (7)	0.0138 (7)
N3	0.0418 (9)	0.0312 (8)	0.0376 (9)	−0.0032 (7)	−0.0069 (7)	0.0060 (7)
N4	0.0206 (6)	0.0277 (7)	0.0248 (7)	−0.0079 (5)	−0.0052 (5)	−0.0006 (5)
C11	0.0393 (10)	0.0217 (8)	0.0430 (11)	−0.0070 (7)	0.0041 (8)	−0.0032 (8)
C12	0.0344 (10)	0.0314 (9)	0.0384 (10)	−0.0093 (7)	−0.0060 (8)	−0.0089 (8)
C13	0.0257 (8)	0.0286 (8)	0.0284 (9)	−0.0055 (6)	−0.0071 (7)	−0.0019 (7)
C14	0.0205 (7)	0.0268 (8)	0.0275 (8)	−0.0030 (6)	−0.0057 (6)	−0.0016 (6)
C15	0.0308 (9)	0.0368 (10)	0.0306 (9)	−0.0044 (7)	−0.0108 (7)	0.0010 (8)
C16	0.0226 (8)	0.0303 (8)	0.0228 (8)	−0.0074 (6)	−0.0063 (6)	−0.0015 (6)
C17	0.0219 (8)	0.0396 (10)	0.0364 (10)	−0.0064 (7)	−0.0077 (7)	−0.0069 (8)
C18	0.0241 (8)	0.0488 (11)	0.0380 (10)	−0.0162 (8)	−0.0032 (7)	−0.0121 (8)
C19	0.0326 (9)	0.0345 (9)	0.0365 (10)	−0.0173 (8)	−0.0034 (8)	−0.0074 (8)
C20	0.0271 (8)	0.0274 (8)	0.0307 (9)	−0.0085 (7)	−0.0048 (7)	−0.0006 (7)

### Geometric parameters (Å, º)

**Table:**

Ag1—N2	2.2305 (14)	C8—C9	1.378 (3)
Ag1—N4	2.2496 (14)	C8—H8	0.9500
Ag1—O1	2.4879 (13)	C9—C10	1.377 (3)
S1—O1	1.4317 (14)	C9—H9	0.9500
S1—O3	1.4331 (14)	C10—H10	0.9500
S1—O2	1.4389 (14)	F3—C11	1.346 (2)
S1—C21	1.821 (2)	F4—C15	1.338 (2)
C21—F6	1.317 (3)	N3—C15	1.310 (3)
C21—F7	1.329 (2)	N3—C11	1.314 (3)
C21—F5	1.329 (3)	N4—C20	1.339 (2)
F1—C1	1.338 (2)	N4—C16	1.344 (2)
F2—C5	1.343 (2)	C11—C12	1.364 (3)
N1—C5	1.311 (3)	C12—C13	1.384 (3)
N1—C1	1.314 (3)	C12—H12	0.9500
N2—C10	1.341 (2)	C13—C14	1.393 (2)
N2—C6	1.345 (2)	C13—H13	0.9500
C1—C2	1.374 (3)	C14—C15	1.384 (2)
C2—C3	1.379 (3)	C14—C16	1.480 (2)
C2—H2	0.9500	C16—C17	1.390 (2)
C3—C4	1.394 (3)	C17—C18	1.380 (3)
C3—H3	0.9500	C17—H17	0.9500
C4—C5	1.385 (2)	C18—C19	1.382 (3)
C4—C6	1.478 (2)	C18—H18	0.9500
C6—C7	1.389 (3)	C19—C20	1.384 (2)
C7—C8	1.372 (3)	C19—H19	0.9500
C7—H7	0.9500	C20—H20	0.9500
			
N2—Ag1—N4	148.65 (5)	C7—C8—H8	120.6
N2—Ag1—O1	124.02 (5)	C9—C8—H8	120.6
N4—Ag1—O1	86.55 (5)	C10—C9—C8	118.71 (19)
O1—S1—O3	115.37 (9)	C10—C9—H9	120.6
O1—S1—O2	114.61 (9)	C8—C9—H9	120.6
O3—S1—O2	115.25 (8)	N2—C10—C9	123.12 (18)
O1—S1—C21	102.70 (10)	N2—C10—H10	118.4
O3—S1—C21	103.67 (10)	C9—C10—H10	118.4
O2—S1—C21	102.70 (9)	C15—N3—C11	115.31 (16)
S1—O1—Ag1	156.68 (10)	C20—N4—C16	118.07 (14)
F6—C21—F7	107.60 (17)	C20—N4—Ag1	119.02 (11)
F6—C21—F5	108.1 (2)	C16—N4—Ag1	121.76 (11)
F7—C21—F5	106.53 (18)	N3—C11—F3	114.28 (18)
F6—C21—S1	111.59 (15)	N3—C11—C12	126.40 (18)
F7—C21—S1	111.55 (15)	F3—C11—C12	119.3 (2)
F5—C21—S1	111.22 (14)	C11—C12—C13	116.10 (18)
C5—N1—C1	115.27 (18)	C11—C12—H12	121.9
C10—N2—C6	118.01 (15)	C13—C12—H12	121.9
C10—N2—Ag1	114.60 (12)	C12—C13—C14	120.70 (17)
C6—N2—Ag1	125.25 (11)	C12—C13—H13	119.7
N1—C1—F1	114.4 (2)	C14—C13—H13	119.7
N1—C1—C2	126.05 (19)	C15—C14—C13	114.98 (16)
F1—C1—C2	119.5 (2)	C15—C14—C16	123.52 (16)
C1—C2—C3	116.39 (19)	C13—C14—C16	121.49 (15)
C1—C2—H2	121.8	N3—C15—F4	114.67 (16)
C3—C2—H2	121.8	N3—C15—C14	126.49 (18)
C2—C3—C4	120.53 (18)	F4—C15—C14	118.84 (17)
C2—C3—H3	119.7	N4—C16—C17	121.95 (16)
C4—C3—H3	119.7	N4—C16—C14	116.22 (14)
C5—C4—C3	115.13 (17)	C17—C16—C14	121.82 (15)
C5—C4—C6	122.25 (16)	C18—C17—C16	119.40 (17)
C3—C4—C6	122.62 (16)	C18—C17—H17	120.3
N1—C5—F2	114.19 (16)	C16—C17—H17	120.3
N1—C5—C4	126.55 (18)	C17—C18—C19	118.87 (16)
F2—C5—C4	119.21 (17)	C17—C18—H18	120.6
N2—C6—C7	121.54 (17)	C19—C18—H18	120.6
N2—C6—C4	117.53 (15)	C18—C19—C20	118.52 (17)
C7—C6—C4	120.92 (17)	C18—C19—H19	120.7
C8—C7—C6	119.7 (2)	C20—C19—H19	120.7
C8—C7—H7	120.1	N4—C20—C19	123.19 (16)
C6—C7—H7	120.1	N4—C20—H20	118.4
C7—C8—C9	118.86 (19)	C19—C20—H20	118.4
			
O3—S1—O1—Ag1	−5.5 (3)	C4—C6—C7—C8	−179.3 (2)
O2—S1—O1—Ag1	131.9 (2)	C6—C7—C8—C9	0.1 (4)
C21—S1—O1—Ag1	−117.5 (3)	C7—C8—C9—C10	1.1 (4)
O1—S1—C21—F6	175.69 (16)	C6—N2—C10—C9	1.5 (3)
O3—S1—C21—F6	55.24 (17)	Ag1—N2—C10—C9	−162.77 (16)
O2—S1—C21—F6	−65.08 (17)	C8—C9—C10—N2	−2.0 (3)
O1—S1—C21—F7	−63.92 (17)	C15—N3—C11—F3	179.02 (17)
O3—S1—C21—F7	175.64 (15)	C15—N3—C11—C12	−0.4 (3)
O2—S1—C21—F7	55.32 (17)	N3—C11—C12—C13	1.2 (3)
O1—S1—C21—F5	54.86 (19)	F3—C11—C12—C13	−178.21 (17)
O3—S1—C21—F5	−65.58 (18)	C11—C12—C13—C14	−0.6 (3)
O2—S1—C21—F5	174.10 (16)	C12—C13—C14—C15	−0.6 (3)
C5—N1—C1—F1	178.08 (19)	C12—C13—C14—C16	178.07 (17)
C5—N1—C1—C2	−1.0 (3)	C11—N3—C15—F4	179.57 (17)
N1—C1—C2—C3	3.0 (4)	C11—N3—C15—C14	−1.1 (3)
F1—C1—C2—C3	−175.97 (19)	C13—C14—C15—N3	1.5 (3)
C1—C2—C3—C4	−2.4 (3)	C16—C14—C15—N3	−177.11 (18)
C2—C3—C4—C5	0.1 (3)	C13—C14—C15—F4	−179.13 (17)
C2—C3—C4—C6	179.92 (18)	C16—C14—C15—F4	2.2 (3)
C1—N1—C5—F2	−179.29 (18)	C20—N4—C16—C17	−0.1 (3)
C1—N1—C5—C4	−1.8 (3)	Ag1—N4—C16—C17	167.60 (13)
C3—C4—C5—N1	2.2 (3)	C20—N4—C16—C14	178.26 (15)
C6—C4—C5—N1	−177.59 (19)	Ag1—N4—C16—C14	−14.0 (2)
C3—C4—C5—F2	179.54 (17)	C15—C14—C16—N4	127.08 (18)
C6—C4—C5—F2	−0.2 (3)	C13—C14—C16—N4	−51.5 (2)
C10—N2—C6—C7	−0.1 (3)	C15—C14—C16—C17	−54.6 (3)
Ag1—N2—C6—C7	162.31 (15)	C13—C14—C16—C17	126.87 (19)
C10—N2—C6—C4	178.53 (15)	N4—C16—C17—C18	0.2 (3)
Ag1—N2—C6—C4	−19.0 (2)	C14—C16—C17—C18	−178.09 (17)
C5—C4—C6—N2	127.75 (19)	C16—C17—C18—C19	−0.4 (3)
C3—C4—C6—N2	−52.0 (2)	C17—C18—C19—C20	0.6 (3)
C5—C4—C6—C7	−53.6 (3)	C16—N4—C20—C19	0.3 (3)
C3—C4—C6—C7	126.7 (2)	Ag1—N4—C20—C19	−167.75 (14)
N2—C6—C7—C8	−0.7 (3)	C18—C19—C20—N4	−0.6 (3)

### Hydrogen-bond geometry (Å, º)

**Table:**

*D*—H···*A*	*D*—H	H···*A*	*D*···*A*	*D*—H···*A*
C10—H10···O2^i^	0.95	2.60	3.239 (2)	125
C13—H13···O2^i^	0.95	2.60	3.173 (2)	119
C13—H13···O3^i^	0.95	2.50	3.400 (2)	159
C19—H19···O3^ii^	0.95	2.53	3.294 (2)	137
C20—H20···O1	0.95	2.55	3.197 (2)	126

Symmetry codes: (i) −*x*+1, −*y*, −*z*+1; (ii) *x*−1, *y*, *z*.
